# Bee pollination improves crop quality, shelf life and commercial value

**DOI:** 10.1098/rspb.2013.2440

**Published:** 2014-01-22

**Authors:** Björn K. Klatt, Andrea Holzschuh, Catrin Westphal, Yann Clough, Inga Smit, Elke Pawelzik, Teja Tscharntke

**Affiliations:** 1 Agroecology, Department of Crop Sciences, University of Göttingen, Grisebachstrasse 6, 37077 Göttingen, Germany; 2 Centre for Environmental and Climate Research, University of Lund, Sölvegatan 37, 22362 Lund, Sweden; 3 Department of Animal Ecology and Tropical Biology (Zoology III), Biocenter, Am Hubland, University of Würzburg, 97074 Würzburg, Germany; 4 Quality of Plant Products, Department of Crop Sciences, Carl-Sprengel-Weg 1, 37075 Göttingen, Germany

**Keywords:** commercial grades, ecosystem services, post-harvest quality, shelf life, strawberry, crop yield

## Abstract

Pollination improves the yield of most crop species and contributes to one-third of global crop production, but comprehensive benefits including crop quality are still unknown. Hence, pollination is underestimated by international policies, which is particularly alarming in times of agricultural intensification and diminishing pollination services. In this study, exclusion experiments with strawberries showed bee pollination to improve fruit quality, quantity and market value compared with wind and self-pollination. Bee-pollinated fruits were heavier, had less malformations and reached higher commercial grades. They had increased redness and reduced sugar–acid–ratios and were firmer, thus improving the commercially important shelf life. Longer shelf life reduced fruit loss by at least 11%. This is accounting for 0.32 billion US$ of the 1.44 billion US$ provided by bee pollination to the total value of 2.90 billion US$ made with strawberry selling in the European Union 2009. The fruit quality and yield effects are driven by the pollination-mediated production of hormonal growth regulators, which occur in several pollination-dependent crops. Thus, our comprehensive findings should be transferable to a wide range of crops and demonstrate bee pollination to be a hitherto underestimated but vital and economically important determinant of fruit quality.

## 1. Introduction

Agricultural production forms one of the most important economic sectors [1]. The quantity of most crop species is increased by pollination [2,3], which is a highly important, but also seriously endangered [4] ecosystem service. More than 75% of the 115 leading crop species worldwide are dependent on or at least benefit from animal pollination, whereas wind and self-pollination are sufficient for only 28 crop species [2]. Thereby, animal pollination contributes to an estimated 35% of global crop production [2]. It is mostly pollination-dependent crops such as fruits that contribute to a healthy human diet by providing particularly high amounts of essential nutrients such as vitamins, antioxidants and fibre [5,6]. Berries especially have been found to benefit human health and are increasingly used for therapies against chronic diseases and even cancer [5]. First attempts for sustaining pollination and other ecosystem services have been aligned in a strategic plan of the Convention on Biological Diversity in Nagoya in 2010. However, recent decisions, such as the new Common Agricultural Policy of the European Union (EU), still endanger ecosystem services by promoting high-intensity agricultural management. Thus, the value of pollination and other ecosystem services is still underestimated or even disregarded in national and international policies. In this study, we expand our knowledge of the underestimated benefits of bee pollination by experimentally quantifying its impacts on crop quantity, quality, shelf life and market value. This should contribute to a better understanding of its monetary and social importance, thereby enhancing a sustainable implementation in future policies. We used strawberries (*Fragaria x ananassa* DUCH.), a crop whose worldwide cultivation is on the increase [1], as a model system.

Strawberry plants flower in several successive flowering periods within a season, with flowers becoming smaller over time [7]. Varieties are self-compatible in most cases, and stigmas become receptive before the anthers of the same flower release pollen, so that allogamy is favoured. Bee pollination increases strawberry weight and shape. Effects depend on varieties [8,9], presumably owing to differences in pollinator attraction [10] and their dependence on cross-pollination [7]. Recent findings about metabolic processes in strawberries support the idea that pollination may also impact shelf life [11–14]. Owing to high fruit sensitivity to fungal infections and mechanical injuries, strawberry fruits have a short shelf life [12]. More than 90% of fruits can become non-marketable after only 4 days in storage [15]. Several studies addressed the potential elongation of the shelf life of strawberries with modified storage procedures [15–19], which highlights how economically important this problem is. Crop features allowing longer storage and thereby, reducing post-harvest losses in supermarkets and households are of major interest worldwide [20], but have so far not been analysed in terms of pollination. Shelf life and pathogenic susceptibility of strawberries are mostly related to fruit firmness [15], but surface colour and sugar–acid–ratios are also involved [15–19]. Fruit colour further determines consumers perception and influences their purchasing behaviour [19], but has never been related to animal pollination. In addition, only few studies report a relation of pollination to firmness [21–23] and sugar contents [22,24–27] of fruits. Hence, comprehensive economic gains of bee pollination are largely unknown and in particular, the potential effect on commercially important parameters of the overall fruit quality has not yet been explored.

We set up a field experiment with nine commercially important strawberry varieties and assessed the influence of self, wind and bee pollination on strawberry fruits using exclusion treatments. We expected that: (i) bee-pollinated fruits would have higher numbers of fertilized achenes, the true ‘nut’ fruits of the strawberry, owing to higher pollination success compared with wind- and self-pollination; (ii) bee pollination would therefore lead to fruits with higher commercial value compared with wind- and self-pollinated fruits, owing to less malformations improving commercial grades and higher fruit weight; as well as (iii) higher firmness and longer shelf life; and (iv) bee-pollinated fruits should have a more intense red colour and lower sugar–acid–ratios, thus improving the post-harvest quality of strawberries.

## 2. Methods

### (a) Experimental set-up

In 2008, we planted nine commercially important strawberry varieties of *Fragaria x ananassa* DUCH. (Darselect, Elsanta, Florence, Honeoye, Korona, Lambada, Salsa, Symphony, Yamaska) on an experimental field. The field was subdivided in 12 plots, and nine rows per plot planted with 18 plants of a single variety per row. All varieties were present in all plots. The sequence of the rows within the plots was randomized. The field was surrounded by two further rows of strawberries to weaken edge effects. Five honeybee hives (*Apis mellifera* L.) and approximately 300 trap nests dominated by *Osmia bicornis* L., have been established for several years close to the field to ensure stable pollination services. Experiments were conducted in 2009 in the first yield year using exclusion treatments on two plants per variety and plot. Following the consecutive flowering periods of strawberries [7], all buds of a plant were covered with Osmolux bags (Pantek, Montesson, France) to allow only self-pollination (self-pollination treatment), gauze bags (mesh width 0.25 mm) to allow self- and wind pollination (wind pollination treatment) or remained uncovered to allow additional insect pollination (bee pollination treatment), respectively. Osmolux bags are semipermeable for water and steam, so that microclimate differences between bagged and unbagged flowers were kept at a minimum. Gauze bags do not create an atmosphere closed from outside the bags and thus have no influence on microclimate. Bags were removed directly after fruit set, when petals began to wither and fall off the flower, and the first approach of a fruit was visible, about 7 days after flower opening. At least 50 fruits per variety and treatment were harvested at maturity. All analyses except the titratable acid content were conducted on the same day of harvesting to avoid influence on post-harvest quality owing to water loss and metabolic procedures.

We collected insect pollinators under favourable weather conditions (*T* > 17°C; low cloud cover; wind speed less than 4 m s^−1^) in 2010, using standardized transect walks. Four strawberry varieties (Honeoye, Elsanta, Korona, Lambada) were selected based on their flowering time, so that all other varieties were flowering at the same time as at least one of these four varieties. Thus, pollinators were collected across the entire flowering season of the commercial strawberry field. Each transect consisted of one row of strawberries per plot of each of the selected varieties. On each of 4 days, four different plots of the experimental field were randomly selected and insects pollinating strawberry flowers were collected using sweep nets on each of the four selected varieties. Each transect was visited for 10 min. Pollinators were identified to species level, and data were pooled across all varieties.

### (b) Commercial value

#### (i) Weight and commercial grades

We calculated the commercial value of each fruit based on fruit weight (BA2001 S, Sartorius, Göttingen, Germany) and the market value of strawberry fruits, which is based on the availability of fruits on the market and commercial grades. Fruits were sorted into commercial grades, owing to aberrations in shape (deformations), colour (areas with yellow or green colour) and size (fruit diameter), following the official trade guidelines [28]. B.K.K. was trained by experienced strawberry growers and colleagues on how to apply the EU trade guidelines. Fruits with no or only slight deformations, with minimal areas of yellow or green colour which did not affect their general appearance, and with a minimum diameter of 18 mm, were sorted into grade extra/one. Fruits showing distinct deformations and larger areas with yellow or green colour, but that had a minimum diameter of 18 mm were classified as grade two. Non-marketable fruits had strong deformations, large areas of yellow or green colour or were of a diameter smaller than 18 mm. Aberrations in colour usually occurred in combination with fruit deformations and were thus not treated separately. Following the above-mentioned Commission's regulation, grades extra and one can be treated separately, but are used combined in practice. We calculated proportions of fruits for each commercial grade and pollination treatment across all varieties (figure 2*b*) and also separately for each variety (see the electronic supplementary material, S2).

We obtained the commercial value of each fruit by multiplying its weight with its market value per gram [29]. The latter was assessed based on harvest time and commercial grades. Harvest time influences the market value of strawberry fruits owing to the availability of fruits on the market: the more fruits that are available, the lower the market value. Thus, fruits that are sorted into lower commercial grades have lower market values. Finally, we extrapolated commercial value to 1000 fruits for a better relationship to market situations.

#### (ii) Firmness and shelf life

We bisected fruits and measured firmness at the centre of each half according to Sanz *et al.* [17] with the following modifications: we fitted the texture analyser (TxT2, Stable Micro System, Surrey, UK) with a 5 mm diameter probe and a 25 kg compression cell, and used a maximum penetration of 4 mm.

### (c) Post-harvest quality

Colourimetric analysis was applied according to Caner *et al.* [19] at two opposite sides of the centre of each fruit in the Lab-colour space using a portable colourimeter (CR-310 Chromameter, Konica Minolta, Badhoevedorp, The Netherlands). The total soluble solids are strongly correlated to the total sugar content of a solution and were measured using a handheld refractometer (HRH30, Krüss, Hamburg, Germany). Measurements for each fruit were conducted twice and repeated when the values differed by more than 0.2 Brix. Fruits were freeze-dried (Epsilon 2-40, Christ, Osterode, Germany), and all samples from the same plant were pooled and milled. To account for an average water content of 82%, which was analysed on a sample of 250 fruits, 0.18 g of each freeze-dried sample was diluted in 20 ml distilled water and titrated according to Caner *et al.* [19].

### (d) Pollination success

We used at least eight fruits from each variety and treatment to analyse the number of fertilized achenes per fruit, which represent pollination success. Each fruit was blended in 100 ml distilled water for two minutes (Speedy Pro GVA 1, Krups, Offenbach, Germany). Fertilized achenes are heavier than water and sink to the bottom, whereas aborted achenes are lighter and accumulate at the water surface. Fertilized achenes were counted (Contador, Pfeuffer, Kitzingen, Germany) after drying for 48 h at 85°C.

### (e) Statistical analysis

In the case of repeated measurements per fruit, we calculated mean values for fruit characteristics. We fitted linear mixed-effects models with random effects allowing treatment slopes and intercepts to vary among varieties [30], using R [31]. To account for space and time errors and unbalance in the data, the random part was completed by two further terms: the interaction of plot, variety and plant, whereas flowering period was included as a crossed random effect. Response variables were commercial value per fruit, fruit weight, firmness, surface colour values (red colour, brightness, yellow colour) and number of fertilized achenes. In the models with sugar–acid–ratio as response variable, only plot and variety were used to complete the random part, because sugar–acid–ratios were calculated based on arithmetic means per plant.

Bee, wind and self-pollination treatments were used as fixed effect levels. To test whether pollination treatments differed and whether there was a main effect of all pollination treatments across all varieties, a model with unpooled treatment levels (full model), models with successively pooled treatment levels and a model without treatment as fixed effect were compared [30] using second order Akaike's information criterion (AICc) and likelihood [32]. This allowed us to test whether all treatments, specific treatment levels only, or none of the treatments had an effect on the response variables. The latter case was taken to indicate that treatment effects were specific to the variety and without a shared common effect between varieties. Residuals were inspected for constant variance, and transformations were used to account for non-normality and heterogeneity, where necessary. Main effect values and parameter estimates were extracted from the model and used for plotting after back transformation.

**Figure 1.**
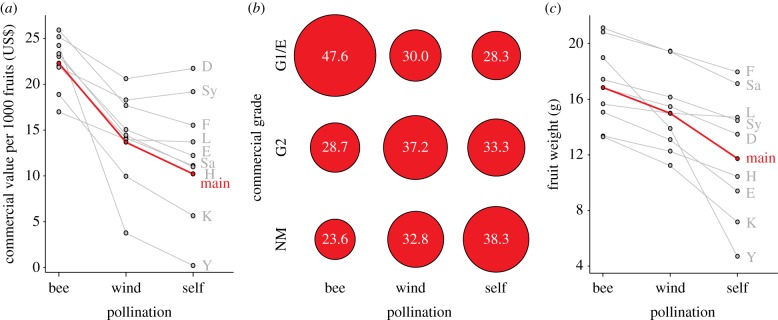
Bee pollination improved the commercial value of strawberry fruits across all varieties as a result of improved commercial grades and higher fruit weight. (*a*) Commercial values for each fruit were calculated in US$, by multiplying fruit weight with the prevailing market value [29], which was assessed due to harvest time and commercial grades. It was extrapolated to 1000 fruits for a better relationship to market situations. (*b*) Commercial grades. Fruit proportions (values within bubbles) were calculated for each commercial grade in dependence on pollination treatments (see the electronic supplementary material, S2 for division into varieties). G1/E, commercial grade one/extra; G2, commercial grade two; NM, non-marketable. (*c*) Weight of strawberry fruits. (*a,c*) Displayed values were extracted from model estimates and back transformed. Grey points display values of varieties (see abbreviations below), red points display the main effect. Lines are shown for better visualization of related points. Solid red lines for the main effect indicated that pollination treatments were stronger than differences between varieties and thus had a main effect across all varieties (see table 2 for AICc and likelihood values). Abbreviations in alphabetical order: D, Darselect; E, Elsanta; F, Florence; main, main effect; H, Honeoye; K, Korona; L, Lambada; Sa, Salsa; Sy, Symphony; Y, Yamaska.

Pearson's chi-squared analysis was used to calculate differences between pollination treatments in the number of fruits for each commercial grade. Differences were shown in proportions for better illustration (figure 1*b*).

## 3. Results

### (a) Commercial value

#### (i) Weight and commercial grades

Strawberry flowers were mainly visited by bees (98.5%). Wild bees were most abundant (64.6%), whereas *A. mellifera* L. (33.9%) and non-bee pollinators (flies: 1.6%) were found less often (table 1). The solitary wild bee *O. bicornis* L. (52.0%) was the most abundant pollinator, whereas other wild bee species accounted for less than 5% of the bee community.

**Table 1.** Pollinators visiting strawberry flowers on the experimental field. (To identify the main pollinators of strawberry flowers on the experimental field, four varieties were randomly selected and insects visiting strawberry flowers were collected. Sweep netting was conducted for 10 minutes on four transects that were randomly selected on each of four different days in 2010. Strawberries were mainly pollinated by solitary wild bees with *O. bicornis* L. being the most frequent species, while honeybees (*Apis mellifera* L.) and non-bee pollinators were less abundant.)

species	abundance	proportion	functional group
*Osmia bicornis* L.	66	52.0	wild bee
*Apis mellifera* L.	43	33.9	honeybee
*Bombus terrestris* L.	5	3.9	wild bee
*Andrena flavipes* Panz.	3	2.4	wild bee
*Merodon equestris* F.	2	1.6	fly
*Andrena gravida* Imhoff	2	1.6	wild bee
*Bombus hypnorum* L.	1	0.8	wild bee
*Bombus lapidarius* L.	1	0.8	wild bee
*Bombus lucorum* L.	1	0.8	wild bee
*Bombus pascuorum* Scop.	1	0.8	wild bee
*Bombus pratorum* L.	1	0.8	wild bee
*Andrena chrysosceles* Kirb.	1	0.8	wild bee
total wild bees	82	64.6	—
total honeybees	43	33.9	—
total non-bees (flies)	2	1.6	—

Bee pollination resulted in strawberry fruits with the highest commercial value (figure 1*a*). On average, bee pollination increased the commercial value per fruit by 38.6% compared with wind pollination and by 54.3% compared with self-pollination. Fruits resulting from wind pollination had a 25.5% higher market value than self-pollinated fruits. Pollination treatments were stronger than differences between varieties and thus had a main effect across all varieties (see table 2 for AICc and likelihood values). Our results suggest that altogether, bee pollination contributed 1.12 billion US$ to a total of 2.90 billion US$ made with commercial selling of 1.5 million tonnes of strawberries in the EU in 2009 [1]—but so far without consideration of the monetary value provided by enhanced shelf life (see below). Price and marketability of strawberries depend on commercial grades of fruit quality (shape, size and colour) [28]. Malformations, in particular, are a common problem affecting strawberry price and marketability [33]. Our experiment showed that pollination treatments significantly differed in the number of fruits for each commercial grade (**χ**_4_^2^ = 60.504; *p* < 0.001, *n* = 1895). Bee pollination reduced malformations and thus enhanced marketability in all varieties except the variety Symphony (figure 1*b*; see the electronic supplementary material, S2 for variety values). The highest proportion of bee pollinated fruits was assigned to the best grade extra/one, whereas non-marketable fruits formed the smallest fraction. By contrast, wind and self-pollination led to high proportions of non-marketable fruits. When compared with wind and self-pollination, bee pollination not only improved fruit shape, but also fruit weight (figure 1*c*). Bee-pollinated fruits were on average 11.0% heavier than wind-pollinated and 30.3% heavier than self-pollinated fruits. Pollination treatments were stronger than differences between varieties and thus had a main effect across all varieties (see table 2 for AICc and likelihood values).

**Table 2.** Delta AICc values and likelihood resulting from model comparisons. (AICc = 0 indicates the model with the highest explanatory power. Lower delta AICc and higher likelihood indicate better explanatory power of a model. Likelihood was calculated for models with delta AICc of less than seven [32]. Best explaining models are highlighted in italics. Sample sizes are given in brackets behind fruit parameters. None, no treatment level pooled; sans, model without fixed effect.)

fruit parameter	pooled levels				
	none	bee = wind	wind = self	bee = self	sans
commercial value (*n* = 1892)					
* *AICc	*0*	4.512	0.173	3.527	2.501
* *likelihood	*0.403*	0.042	0.370	0.069	0.115
fruit weight (*n* = 1895)					
* *AICc	*0*	4.162	3.507	4.872	3.137
* *likelihood	*0.627*	0.078	0.109	0.055	0.131
shelf life (*n* = 1268)					
* *AICc	*0*	0.347	1.791	7.218	5.273
* *likelihood	*0.431*	0.362	0.174	—	0.031
red colour (*n* = 1279)					
* *AICc	1.428	1.608	*0*	2.021	0.323
* *likelihood	0.155	0.142	*0.317*	0.115	0.270
sugar–acid–ratio (*n* = 345)					
* *AICc	2.128	3.244	*0*	1.247	1.147
* *likelihood	0.131	0.075	*0.378*	0.203	0.213
pollination success (*n* = 356)					
* *AICc	*0*	4.267	9.192	8.704	7.290
* *likelihood	*0.894*	0.106	—	—	—

#### (ii) Shelf life

**Figure 2.**
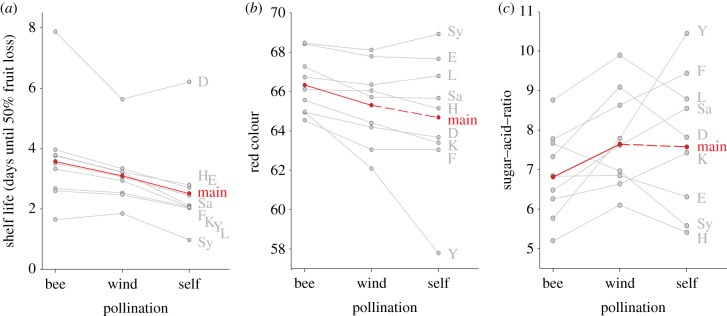
Bee pollination improves the post-harvest quality of strawberries. (*a*) Shelf life in days until 50% fruit loss was calculated from firmness values that were related to published data on firmness decreases during storage (see the electronic supplementary material, S3 for calculations) [15]. (*b*) Red colour intensity. (*c*) Sugar–acid–ratios. Red lines for the main effect are dashed when pollination treatments did not differ, indicating stronger variety effects than pollination treatments (see table 2 for AICc and likelihood values). Further details and abbreviations are explained in the caption of figure 1.

Bee pollination strongly impacted the shelf life of strawberries by improving their firmness (figure 2*a*). The firmness values of each treatment and variety were related to shelf life, measured as the number of days until 50% of fruits had been lost owing to surface and fungal decay (see the electronic supplementary material, S3). Higher firmness resulting from bee pollination potentially elongated the shelf life of strawberry fruits by about 12 h compared with wind pollination, and by more than 26 h compared with self-pollination. After 4 days in storage, only 29.4% of the wind-pollinated fruits and none self-pollinated fruit were still marketable, whereas, at the same time, 40.4% of the bee-pollinated fruits remained in a marketable condition. Thus, bee pollination accounted for a decrease of at least 11.0% in fruit losses during storage. These findings suggest that the value for bee pollination calculated in §3a(i) has to be increased to accommodate this impact on the shelf life of strawberries. Hence, pollination benefits on the shelf life of strawberries potentially added another 0.32 billion US$ to the commercial value of strawberry pollination (without shelf-life effects: 1.12 billion US$). In total, bee pollination contributed 1.44 billion US$ to a total of 2.90 billion US$ made with the commercialization of 1.5 million tonnes of strawberries in the EU in 2009 [1]. Pollination treatments had a main influence on shelf life across all varieties (see table 2 for AICc and likelihood values). Varieties producing fruits with high firmness benefitted most from bee pollination.

### (b) Post-harvest quality

In most varieties, bee-pollinated fruits had a more intense red colour compared with fruits resulting from wind and self-pollination (figure 2*b*). Self-pollinated fruits of the varieties Lambada and Symphony showed the most intense red colour in the self-pollination treatment. The bee pollination treatment differed from the two other pollination treatments across all varieties, whereas strong variety differences imped a difference between wind and self-pollination treatments (see table 2 for AICc and likelihood values). The brightness of bee- and wind-pollinated fruits was similar and highly correlated to yellow colour intensity (see the electronic supplementary material, S4 and S5). Both colour properties did not differ between bee and wind pollination, but self-pollinated fruits were darker and had less intense red colour. Thus, bee pollination resulted in bright fruits with a more intense red colour than wind pollination fruits, whereas self-pollinated fruits were darker and less red (figure 2*b* and the electronic supplementary material, S4).

Senescence of strawberries is not only related to losses in firmness and colour changes, but also to increasing sugar–acid–ratios. Bee-pollinated fruits generally had a lower sugar–acid–ratio compared with wind- and self-pollinated fruits across all varieties (figure 2*c*), but fruits of the varieties Elsanta and Symphony had a higher sugar–acid–ratio with bee pollination. The difference between wind and self-pollination remained variety-dependent (see table 2 for AICc and likelihood values), whereas the sugar–acid–ratio of fruits resulting from bee pollination differed from both other treatments across all varieties.

### (c) Pollination success

**Figure 3.**
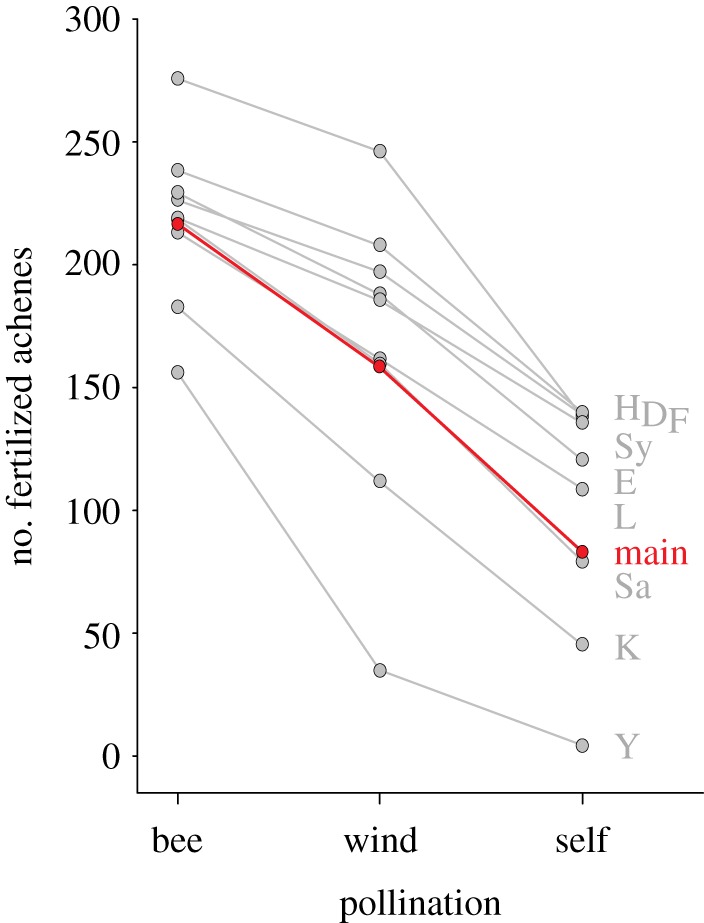
Bee pollination had higher pollination success, calculated as the number of fertilized achenes per fruit (see table 2 for AICc and likelihood values). Further details and abbreviations are explained in the caption of figure 1.

Pollination success was related to the number of fertilized achenes dependent on pollination treatments. Bee pollination was much more efficient than wind and self-pollination, resulting in a higher number of fertilized achenes per fruit across all varieties (figure 3; see table 2 for AICc and likelihood values). Bee pollination on average increased the number of fertilized achenes by about 26.8% compared with wind pollination and about 61.7% compared with self-pollination. Wind-pollinated fruits had a 47.7% higher number of fertilized achenes than fruits resulting from self-pollination. This confirms our findings to be true effects of bee pollination.

## 4. Discussion

We found bee pollination, which was mainly conducted by solitary wild bees, to play a key role for several features of the quantity and quality of strawberry fruits. Bee pollinated fruits showed less malformations, greater fruit weight and longer shelf life, resulting in higher commercial value as well as improved post-harvest quality by more intensive red colour and lower sugar–acid–ratios than fruits resulting from wind and self-pollination.

The mechanism behind the benefits of strawberry pollination by bees is based on the fertilization of the true ‘nut’ fruits of the strawberry, the achenes [11–14]. During their visits, bees allocate pollen homogeneously on the receptacles, increasing the number of fertilized achenes per fruit [34]. While unfertilized achenes resulting from insufficient pollination have no physiological functionality, fertilized achenes produce the plant hormone auxin [35], which mediates the accumulation of gibberellic acid [14]. Together, these plant hormones induce fruit growth by improving cell progeny and size, thereby enhancing the weight of strawberry fruits [12]. This further improves fruit quality and thereby commercial grades [12] by preventing malformations, which are caused by areas of unfertilized and thus physiologically inactive achenes [33].

How can pollination induce a longer shelf life in strawberries? The shelf life of strawberries and other fruits is mostly dependent on their firmness [15,33], which is also functionally based on fertilized achenes [33] and thus dependent on successful pollination. Auxin and gibberellic acid delay fruit-softening and thereby enhance firmness and shelf life, by limiting the expression of several fruit-softening proteins, the so-called expansins [11]. Higher levels of both plant hormones also increase the post-harvest quality of strawberries. Although auxin alone reduces the accumulation of anthocyanins [11], high levels of both auxin and gibberellic acid can, in conjunction, increase anthocyanin accumulation [12]. In contrast to firmness and colour changes, sugar–acid–ratios of strawberries are not directly affected by auxin and gibberellic acid [12]. But higher firmness of fruits is associated with more stable cell walls which might reduce respiration, which is known to limit metabolic processes affecting sugar and acid contents during storage [19]. Indirect positive effects of pollination are therefore probable.

Plant hormones that can influence the quality of fruits and vegetables are known to occur not only in strawberries, but also in several other crops [36] that require animal pollination [7]. Crops such as coffee [37] and blueberry [38] benefit from animal pollination in terms of fruit set and fruit size; and it has been shown elsewhere that fruit shape can benefit from increased animal pollination [7]. This indicates that our findings may be transferable to a high variety of crops and that animal pollination may largely contribute to crop quality. However, only few studies have focused to date on effects of pollination other than the effects on crop yield and fruit set. It has been shown that the sugar content of loquats [24,26], vine cactus [25] and oriental melon [22] as well as the firmness of oriental melon [22] and cucumber [23] can be increased by animal pollination. Contrasting results are available for the tomato, whereas Al-Attal *et al.* [21] showed that pollination increased the firmness of tomatoes in greenhouses, but pollination had no effect on the firmness of cherry tomatoes under field conditions [27]. Oilseed rape is another important crop whose quality benefits from insect pollination by higher oil content and lower chlorophyll content [39]. These results support the assumption of a general impact of pollination on multiple aspects of crop quality. However, such comprehensive findings about the benefits of pollination on crop quality, yield and commercial value as in our study, which can be mechanistically well linked to formerly reported physiological processes, have never, to our knowledge, been reported before.

Our results showed strawberries to be almost exclusively visited by bees, with solitary wild bees being most abundant. This contrasts with earlier findings, where honeybees were the most common pollinators of strawberries and other crops [7] and further shows that the wild bee pollination can be important for crop production, if wild bees are abundant close to crop fields. Wild bee pollinators have already been shown to be effective crop pollinators [40], including strawberries [41]. Additional experiments are required to assess the current abundance of wild bee pollinators and thus their importance for strawberry production on commercial strawberry fields under conventional management conditions.

In our study, we used an innovative approach to the calculation of the commercial value of pollination by considering not only overall yield [2,3] but also crop quality in terms of trade classes, shelf life and changing market values. Shelf life is a major factor determining the commercial value of pollination. Globally, between one-third and a half of all fruits and vegetables are lost due to mechanical damage and deterioration during handling, transport and storage directly after harvest, or wasted at retailer and consumer levels [42]. This illustrates the commercial and social importance of crop shelf life and the far-reaching impact of pollination deficits.

Of course, our calculations may still underestimate the commercial value of bee pollination as they are not considering commercial pollination benefits related to colour, sugar–acid–ratio and other taste components.

## 5. Conclusion

In conclusion, our results showed that crop pollination is of higher economic importance than hitherto thought. Plant hormones, the production of which is mediated by pollination, occur in several other pollination-dependent fruits and vegetables [36]. This highlights the major importance of animal pollination for crop quality in other crops in addition to strawberries. Quality improvements of crops can greatly affect marketability and contribute to reducing food loss and waste. In the industrialized countries, 30–50% of all crops are thrown away at retail and consumer levels [20,42]. Under the current scenario of rapid human population increase and global food demand [43], achieving high quality and quantity of crops is a pressing issue. Our study suggests that comprehensive analyses of the benefits of pollination for animal-dependent crops, which comprise 70% of all major crop species [2], may clearly increase estimates of the economic value of this ecosystem service. Pollination appears to be economically much more important than previously recognized and needs better support through adequate agricultural management and policy.
